# Household Survey of Pesticide Practice, Deliberate Self-Harm, and Suicide in the Sundarban Region of West Bengal, India

**DOI:** 10.1155/2013/949076

**Published:** 2013-10-09

**Authors:** Sohini Banerjee, Arabinda Narayan Chowdhury, Esther Schelling, Mitchell G. Weiss

**Affiliations:** ^1^Institute of Psychiatry, 7 D.L. Khan Road, Kolkata 700 025, India; ^2^Stuart Road Resource Centre, Northamptonshire Healthcare NHS Foundation Trust, Corby, Northants NN17 1RJ, UK; ^3^Department of Epidemiology & Public Health, Swiss Tropical & Public Health Institute, Basel, Switzerland; ^4^University of Basel, Basel, Switzerland

## Abstract

The toxicological impact and intentional ingestion of pesticides are major public health concerns globally. This study aimed to estimate the extent of deliberate self-harm (DSH) and suicides (suicidal behaviour) and document pesticide practices in Namkhana block of the Sundarban region, India. A cross-sectional study was conducted in 1680 households (21 villages) following a mixed random and cluster design sampling. The survey questionnaire (Household Information on Pesticide Use and DSH) was developed by the research team to elicit qualitative and quantitative information. The Kappa statistic and McNemar's test were used to assess the level of agreement and association between respondents' and investigators' opinions about safe storage of pesticides. Over five years, 1680 households reported 181 incidents of suicidal behaviour. Conflict with family members was the most frequently reported reason for suicidal behaviour (53.6%). The Kappa statistic indicated poor agreement between respondents and investigators about safe storage of pesticides. The pesticide-related annual DSH rate was 158.1 (95% CI 126.2–195.5), and for suicide it was 73.4 (95% CI 52.2–100.3) per 100,000. Unsafe pesticide practice and psychosocial stressors are related to the high rates of suicidal behaviour. An intersectoral approach involving the local governments, agricultural department and the health sector would help to reduce the magnitude of this public health problem.

## 1. Introduction

It has been estimated that annually about 5 billion pounds of pesticides are used globally in agriculture. More than two decades ago the World Health Organisation (WHO) estimated 3,000,000 people were hospitalised for pesticide poisoning each year throughout the world, two-thirds due to intentional poisoning and 7.3% of the total number resulting in mortality [1, 2]. Since the publication of this report, a number of studies have indicated that suicidal behaviour, including non-fatal deliberate self-harm (DSH) and suicides, particularly with pesticide are serious global public health problems in many low- and middle-income (LAMI) countries [3–8].

A review [9] indicated that most epidemiological research on DSH and suicide with pesticides in LAMI countries are based on hospital or clinical data. Such clinical data, however fail to consider many aspects of the problem associated with pesticide use in the community. Hospitalisation of patients with intentional pesticide ingestion depends on a number of factors, including access to treatment, seriousness of the attempt [10], gender, social stigma, and the type of poison ingested. Clinic-based data tell only part of the story; they typically summarise demographic features of cases, and sometimes psychiatric and medical risk factors. It rarely considers contexts, motivations, or the easy availability of means for suicidal behaviour (both non-fatal DSH and fatal suicides). Community studies are needed in order that complementary community and hospital studies may guide strategies for suicide prevention [11–14].

In the course of developing community mental health research in the Sundarban region of India, various segments of village communities expressed concerns about suicidal behaviour, focusing on pesticide ingestion [15–17]. Acknowledging the problem and responding to the requests from the community, a programme for preventing suicidal behaviour in the region that combined research, clinical services, and community interventions was developed.

As a part of this effort, a household survey was conducted in 1,680 households on an island of the Sundarban region. This cross-sectional study assessed household reports of pesticide practices and use of pesticides and other methods for suicidal behaviour in the community. The survey also assessed accidental poisoning and the level of awareness among farmers about the safety, storage, and ill-effects of pesticide use. 

## 2. Methods

### 2.1. Study Setting

**Figure 1:**
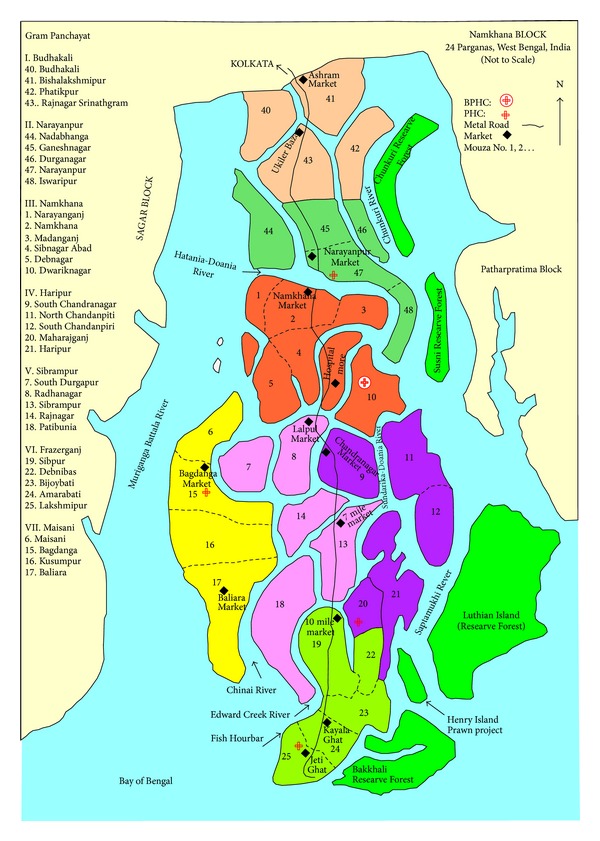
Map of Namkhana showing the 21 study villages.

Since the partition of the Indian subcontinent in 1947, one-third of the Sundarban region lies in India and the rest in Bangladesh and it is the is the largest tidal mangrove delta of the world [18, 19]. The Indian Sundarban region is located at the southernmost tip of the state of West Bengal. On the west it is bounded by the Hoogly river, on the east by the Ichamati-Kalindi-Raimangal rivers, on the south by the Bay of Bengal, and on the north by the imaginary Dampier-Hodges line (The Dampier-Hodges line is an imaginary line which was drawn by two colonial surveyors in 1822. It indicates the northern-most limits of estuarine zones affected by tidal fluctuations) [20]. The region comprises both island and mainland community development blocks (CDBs), which are the lowest level of administrative units of a district in a rural region. Namkhana is one of the island blocks of the Sundarban region. It is situated 105 kms south of the state capital, Kolkata, and covers an area of 227 square kilometres. In 2001, the total population of the region was 160,630 [21]. Seven gram panchayats (GPs), or local self-government organisations govern 34 villages of Namkhana block. Two rivers, the Hatania-Doania and Chinai, trisect the region into three distinct geographical units. For this study three villages from each of the seven GPs were selected to represent a range of ecological and demographic conditions within the administrative block of Namkhana (Figure 1). The villages were Budhakhali GP (40,41,43), Narayanpur GP (44,45,46), Namkhana GP (1,4,10), Haripur GP (9,11,12), Sibrampur GP (7,8,13), Fraserganj GP (22,23,24) and Maisani GP (15,16,17).

### 2.2. Sample Size Calculation

Prior to this study, no community research on suicidal behaviour had been conducted in this area. Absence of prior knowledge of sampling parameters made the sample size computation for a community survey with responses on a sensitive issue as DSH and suicide difficult. In order to guide sample size calculation and to pre-test the survey instrument a pilot survey was conducted in all the households (*n* = 214) in Lakshmipur Abad, a village presenting ecological and demographic characteristics similar to the other villages of Namkhana [22]. The primary purpose of the instrument was to gather information about any events of non-fatal DSH carried out by any of the family members within the past 5 years that were known to the informant. People who died as a result of a DSH event were classified as suicides. Poisonings of children less than ten years of age were considered to be accidental poisoning. Recall period was 5 years. Fifteen (7.01%) households reported DSH cases in the pilot study.

Considering the prevalence rate from the pilot study, with a ±2% precision, and setting the confidence interval at 99.9% the sample size was computed to be 1680 households. The study universe was 30,000 households distributed among 7 GPs of Namkhana block. A mixed multistage random and cluster design was followed for the purpose of the survey on DSH from the households. A two-stage cluster sampling technique regarded villages as the first cluster and households as the second clustering unit. Household was defined as people sharing a common kitchen. These households were drawn up from the Household Register of each village, which was collected from the Block Development Office (BDO), and numbers were assigned to households. Three villages were randomly selected from each GP. Thus, 21 of the 34 villages of the Namkhana block were selected randomly. This was done to allow equitable representation to each GP. Distributing 1680 households among the 21 selected villages required 80 households to be interviewed per village, and these households were selected from a complete list of all households using computer generated random numbers. For every village, extra 25% households were generated from the random list to provide substitutes for unavailable households. The study design thus reduced to a mixed random and cluster design with the household as the study unit.

The pilot study indicated that agriculture was carried out mostly by men but women who were involved had a more passive role in using pesticides and offered very little information on pesticide practice. Women, in the region were mostly not allowed to be involved with pesticides because they are considered to be physically unfit to handle pesticides and the community expressed concerns about the effect of the pesticides on the reproductive health of women. Thus, it was decided that only an adult male (minimum 18 years) would be interviewed for the household survey. If, during the actual household survey, a selected household had no adult male member, the household would be skipped and the next household on the random number list would be considered for survey.

### 2.3. Survey Instrument and Data Collection

A survey schedule (Household Information on Pesticide Use and DSH) was designed to elicit qualitative, and quantitative information of agricultural practices, pesticide use, and accidental poisoning and DSH from the study households. The 14-item questionnaire began with a short introduction describing objectives of the study and purpose of the interview, followed by questions about demographic information of respondents, including age, level of education and occupation of the informant. The first three questions pertained to land holding and agricultural practice of the household. Questions 4–11 addressed the issue of household chemical use, pesticide use, pesticide storage, respondents' and investigators' opinions about safe pesticide storage and type of shop from which it was purchased and knowledge about ill-effects of pesticide on crops and human health. The investigators' criteria for safe storage of pesticides were if they were kept in a locked box and in a confidential place known only to the respondent or household head and out of reach of both children, and other members of the family. The last three items focused on accidental poisoning and suicidal behaviour with pesticides and with other agents. These three questions further aimed to clarify the sex, age, hospitalisation, outcome, and reasons of persons indulging in suicidal behaviour. Recall period of any self-harm event was 5 years. There was an additional section for investigator to record personal comments. Informed consent was obtained before each interview. The instrument was pretested during the pilot survey and altered based on experience and recommendations from the community. The modified instrument was used for the main survey. Data were collected from May 2004 to April 2005 by the first author (SB) and six research assistants who were supervised by the first and second authors (SB, ANC).

### 2.4. Data Analysis

Data were entered in Microsoft Access and analysed with Stata (Intercooled Standard version 8.0). Descriptive analysis of various variable such as age, level of education, primary occupation, types of crops cultivated, household chemical use, pesticide use, pesticide storage, knowledge about the ill-effects of pesticide on crops and on health, and events of suicidal behaviour. While the Kappa statistic was computed to measure the level of agreement between the respondents and the investigator about the safe storage of pesticide, the McNemar's Test was used to assess the association between the groups of responses. The analysis was done on a subset (*n* = 1221) of the study population who stored pesticides. Community rates of DSH and suicide with pesticide exclusively and all means, including pesticides, were calculated per annum considering that the DSH and suicides occurred constantly over a period of 5 years. The total surveyed population comprised all members of the enrolled 1680 households. The total female and male population were calculated from this total using the proportions of the census data of West Bengal, 2006.

## 3. Results

A total of 1680 households (10627 members) were surveyed in the 21 villages of Namkhana Island. The median age of the respondents was 42.5 (range 18–90 years). Most (44.5%) of the respondents had secondary level (standard V to standard XII) education followed by 26.8% of the respondents who had primary education (standard I to standard IV); 6.0% of the respondents reported that they had education higher than secondary level and the rest had no education. Respondents often had more than one occupation. They could specify a primary occupation, based on their principal source of earning. The three primary occupations reported most frequently were farming (41.5%), daily labour (22.1%), and fishing (16.4%).

A total number of 1,236 households (73.6%) reported possessing agricultural land. Most households cultivated more than one crop. The crops commonly grown by the households were paddy (rice, 81.7%) chilli (*Capsicum annuum*) (48.3%), betel leaf (*Piper betle*) (20.8%), and vegetables. A few households also cultivated watermelon and sunflower.

All households reported using kerosene, for lighting, since electricity was still not available in most villages and kerosene lamps were the main source of light. The majority of the households used pesticides for agricultural purposes (72.7%) and 31.5% of the 444 households not possessing agricultural land reported using pesticides. Of the households using pesticides, 46.3% of the households reported storing pesticides inside the house and only 8.2% households did not store pesticides but used it immediately after purchase. Pesticides were stored outside the house by 22.4% households while 70 (5.7%) households kept them both inside and outside the house. An overwhelming majority (98.0%) of the farmers reported spraying pesticides without protective gears such as gloves and boots.

Over a period of five years, a total of 169 households (9.9%) reported 181 incidents of suicidal behaviour, of which 136 were DSH (75.1%) and the rest were suicides. The most commonly used methods in suicidal behaviour were pesticides (68.0%) followed by indigenous poisons (18.2%), hanging (8.3%), burning (2.2%), and other methods. Pesticides were the most frequently reported method adopted for both DSH and suicide. Hospitalisation was done on 108 occasions (59.7%), most of which were for pesticide ingestion (72.2%), 25.0% for indigenous poisons, only three for burning, and one for hanging. The two most frequently reported reasons for suicidal behaviour were quarrel with spouse (53.8%) and other family members, including in-laws, parents, sibling, and children (19.0%). Various issues, ranging from extramarital relations and physical abuse to parental retribution for smoking, were identified as the various dimensions of family conflict by the community. The associated problems of alcohol abuse were also mentioned in a few instances.

Table 1 shows the distribution of pesticides storage inside and outside the household. Respondents mainly expressed concerns about the safety of children and thus more than three-quarters of the households stored pesticides in places that were out of reach of children. Only 27.3% of the households had provisions for storing pesticides in a locked box and 29.3% in a confidential place. Here, the term “confidential” was used to indicate a place which only the person primarily engaged in agriculture was aware about. Majority (61.9%) of the respondents who reported storing pesticides outside the household stored it in the agricultural field, under the soil. One person reported storing it on a tree beside a neighbour's pond. He expressed concerns about storing it in the field, fearing theft. He said that it was too risky to keep it in the field and devised his own way of storing the pesticide. At the same time he expressed concerns about storing it at home.

Table 2 shows the level of agreement between respondent and investigator regarding safety of pesticide storage. Of the 1221 respondents, 1074 (87.9%) judged their storage arrangement to be safe, but, the investigator considered fewer of these to be safe. Only on three occasions did the investigator consider storage to be safe when the respondent perceived it otherwise (0.6%). There was poor agreement between investigator and respondent on 1221 responses on pesticide storage (*κ* = 0.16). The McNemar's test indicated there was an association between the responses of the respondent and the investigator. There was a bias in the sense that the investigator tended to agree more with the respondents on the issue of unsafe storage of pesticides and inclined to disagree with the farmers who thought their pesticides were stored safely.

Of the 1221 households interviewed, only a little more than a quarter had any information about the ill-effects of pesticide use on crops. In comparison to the households' knowledge about the ill-effects of pesticide use on crops, they were more aware about its adverse impact on health (37.0%). They gathered information primarily through their own experiences and from other farmers. The agriculture department and GPs played very little role in the dissemination of information about the side effects of pesticide use (Table 3).

Farmers reported that the block agricultural department assigned an agricultural advisor for each GP, who is commonly referred to as KPS (*Krishi Prayukti Sahayak*), but he provided no assistance. The KPS is supposed to visit each village twice a month to inform farmers about newer methods of cultivation in order to increase crop production, answer their queries and promote safe pesticide practice. However, he is rarely to be seen and as a farmer summed up:

*“I have been cultivating for the last 20 years. Earlier, the KPS used to visit us regularly but since the last 5 to 10 years, he is rarely to be seen. The only day he is around is when he has to collect his salary from the block agricultural office at the end of the month.”*

Another finding was related to the awareness of danger in larger doses but failure to appreciate the risk of small doses or exposure to pesticide.

The overall annual rates for DSH and suicide in Namkhana were very high. The suicide rate was eight times higher than the national average of 10.6 per 100,000. The pesticide-related annual DSH was 158.1 per 100,000 (95% CI 126.2–195.5) and suicide rate was 73.4 per 100,000 (95% CI 52.2–100.3). Both DSH and suicide rates were higher in women than in men (Table 4).

## 4. Discussion

This community based epidemiological study highlighted four important findings, notably the issue of pesticide storage; lack of knowledge about safe pesticide practice; the interactions between pesticide practice and suicidal behavior; the high DSH and suicide rates in Namkhana CDB of the Sundarban region.

### 4.1. Pesticide Practice

**Figure 2:**
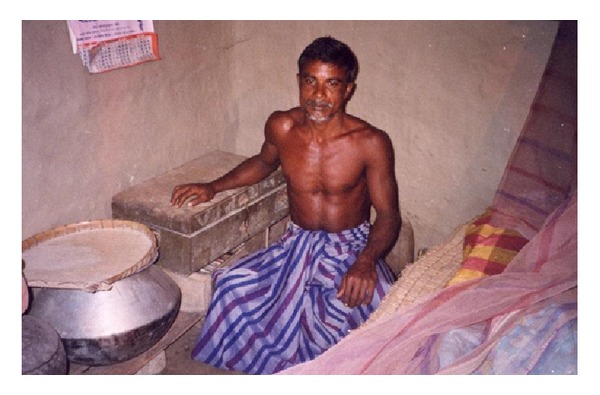
Pesticide stored in cooking vessels and in trunks (with clothes) inside the bedroom (Dwariknagar village).

**Figure 3:**
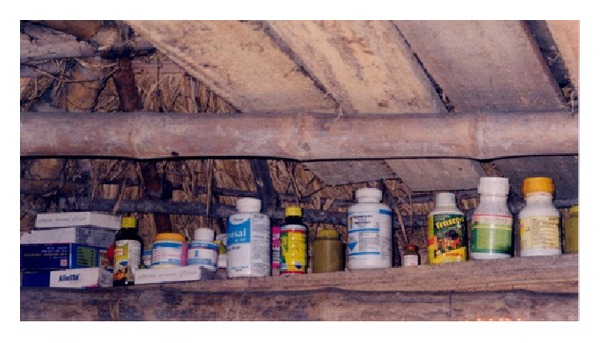
Pesticide kept under the roof in living room (Bagdanga village).

**Figure 4:**
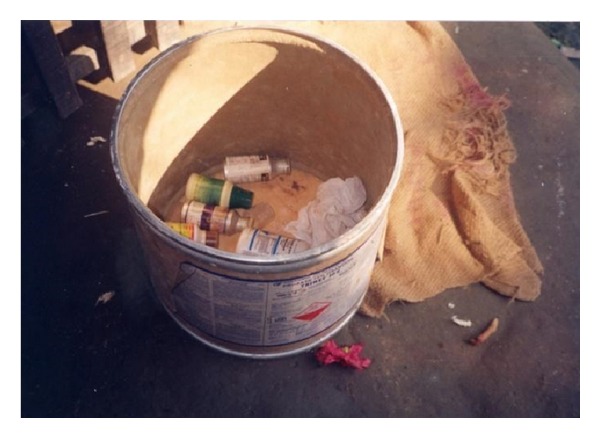
Pesticide in open container inside the cattle-shed (Amarabati village).

Most households stored pesticides at home and in a way that was considered to be safe by the respondents but unsafe in the opinion of the investigator. Unlike in industrialised countries, most farmers in low- and middle-income countries cultivate small areas of land and live in a single or two roomed huts, and this is true also for farmers in Namkhana. Most farmers do not have the financial capacity to build a separate room to store pesticides. Thus, they are compelled to store it either in the living quarters or in the agricultural field. Studies in Sri Lanka and China have reported farmers store their supplies of pesticides within or near the household [3, 23]. However, most farmers preferred to keep the pesticides at home, in cartons, open shelves in the wall, or tucked away in a tile on the roof (Figures 2, 3 and 4). The reasons they cited were that most farmers were poor and some of them could not afford cupboards or locked boxes. Pesticides are expensive and hiding it in the agricultural field was considered to be unsafe as they may be stolen. Furthermore, rain water could seep into the container and render the pesticide unusable. While they expressed personal concerns about storing them in the fields, they totally ignored the environmental hazards such as soil contamination from spillage.

The farmers considered pesticides to be stored in a safe manner if they were kept out of reach of children but with little regard for the safety of other members of the family. Farmers added that it was impossible to keep pesticides in locked boxes or in a confidential place out of reach of other members of the family because they were sometimes actively involved in agricultural activities. Moreover, some farmers who were also involved in fishing said that another member of the family had to spray pesticides while they were away, and they had to know where the pesticides were stored. Although, most farmers are poor and cannot afford to purchase separate cupboards or locked boxes for storing pesticides, those who did have cupboards or locked boxes stored their pesticides along with other belongings such as clothes. Some farmers mentioned that, during the farming season, they are frequently required to spray pesticides, and it is inconvenient for them to store pesticides away in locked cupboards or boxes. These factors argued against safe pesticide storage recommended by national and, international agencies

### 4.2. Lack of Knowledge about Safe Pesticide Practice

**Figure 5:**
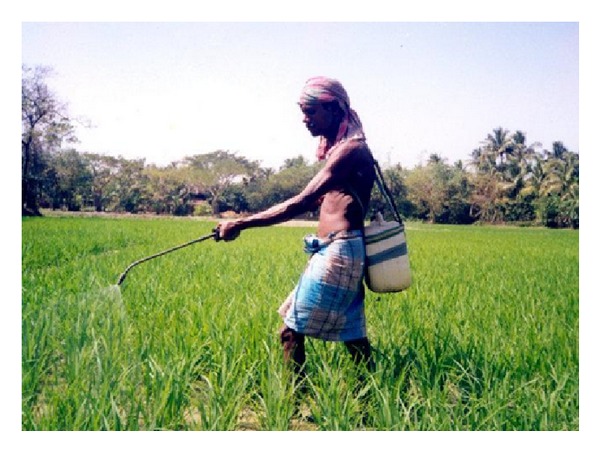
Farmers spraying pesticides in paddy field without protective gear (Namkhana village).

**Figure 6:**
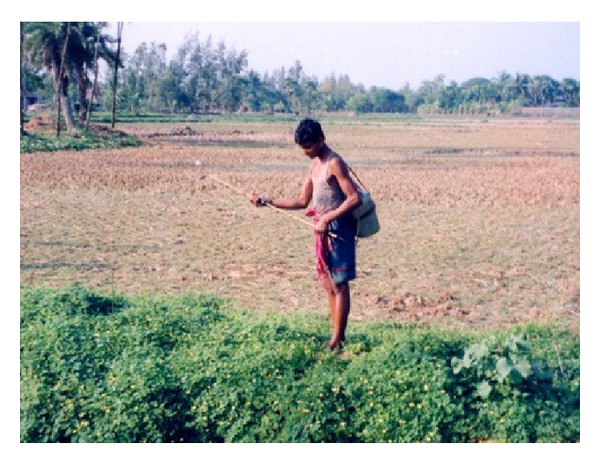
Farmer applying pesticides in vegetable field without protective equipment (Haripur village).

Most respondents reported that they were unaware of the ill-effects of pesticide use, either on health or on the environment. For those who did report knowledge about the negative effects of pesticide use, knowledge came primarily from their own experience or those of fellow farmers. They openly declared that pesticide shop owners and aggressive advertising by pesticide companies highlighted only the positive impact of pesticide use, but, not their harmful effects. The agricultural department and the GPs in Namkhana had little or no role in educating the farmers about safe pesticide practice. Similar to findings from other rural settings in India [24], this study too observed that farmers did not adorn preventive apparel (wearing garments, gloves, protective footwear, etc.) while spraying pesticides (Figures 5, and 6). Though many of farmers reported experiencing physical discomfort while using pesticides such as symptoms of nausea, irritation in the eyes and skin, they did not seek medical help. A common notion the farmers held was that drinking tamarind water would relieve nausea. A few farmers exhibited a nonchalant attitude when they declared that they opened the pesticide container with their mouth and some of them said they tasted it before applying it on the plants. These findings reflect their inadequate knowledge of pesticide hazards and the need to promote awareness of safe pesticide practice and storage. Studies on farm workers' knowledge about the effect of pesticide use conducted in Florida, North Carolina, USA, and Nueva Ecija, Philippines, Egypt, Turkey, and Malaysia reported similar findings [25–29]. 

### 4.3. Pesticide Practice and Suicidal Behaviour

This study, similar to other studies found that patients using pesticides for DSH were taken to the hospital while those using other methods were not [9, 30, 31]. Reliance on clinical data alone may overestimate DSH with a particular method, pesticides, as found in this study while unerringly overlook the influence of other methods used in DSH and suicide. Hence, a community study, juxtaposed with clinical research on DSH, may yield a more complete picture of the problem in a community and thereby help in designing an effective intervention to prevent suicidal behaviour.

This study found quarrel with spouse and other family members prompted the suicidal behaviour. There is a need to sensitise the local community about the typical psychosocial contexts in which suicidal behaviour occur and to encourage community support to assist those who are vulnerable to involve themselves in suicidal behaviour. The insights developed during the course of this community survey help to understand the local context and situation which need to be taken into account in order to design an effective strategy for DSH and suicide prevention suited to the particular needs of the community [15].

### 4.4. High Rates of DSH and Suicide

Few studies have identified rates of non-fatal DSH either globally or at a national level. This study has made one of the earliest efforts to document non-fatal DSH rates in an Indian community. This study reports high rates of female suicide, higher than their male counterparts [15, 32, 33]. This finding is contrary to global findings where more men die by suicide and more women attempt DSH [34]. This may have some relation to the fact that the disadvantage of female gender roles contributes to the vulnerability of women in low- and middle-income countries, particularly young married women. From a very young age, the patriarchal systems in low- and middle-income countries inculcate in women the belief that they are submissive, docile, timid, and in general, subordinate to men within and outside the household.

Traditional Indian marriages require a new bride to live with her husband's family, especially in rural areas. She is expected to take on numerous responsibilities and is often held responsible and blamed for conflicts within the household. Amidst the hostile environment they feel helpless and fear losing their husband's sympathies. They opt for DSH as a way of putting an end to psychological pain and misery [33, 35]. Findings from this study indicate a serious problem confronting the society in Namkhana, as in many low- and middle-income countries, namely, gender-based inequality. To address this issue and bring about a change in the social position of women in Namkhana require initiatives in various spheres of life. Strengthening legislative measures in favour of women, education, and developing better coping skills when faced with negative life situations are just a few ways by which this may be achieved.

The findings of this study are also contrary to global trends, which consider DSH to be 10 to 20 times more common than suicide [36]. DSH events as reported in this study were approximately 4 times more than suicide rates. Further research is warranted on this issue in order for this finding to be generalised.

## 5. Limitations of the Study

The rates of suicidal behaviour have to be interpreted with a certain degree of caution considering that the recall period of DSH and suicide was 5 years. It was not possible to crosscheck the information on suicidal behaviour provided at the household level. However, this limitation will continue to exist in view of the socio-economic and educational canvas of the population. The interpretation pertaining to storage of pesticides by the respondent was subjective which lead to a difference with regard to the assessment by the interviewer. As a result the extent of agreement which was also calculated was poor. The assessment was nevertheless based on recommendations for safe storage, and it was usually clear, rather than ambiguous, lending validity to the findings.

## 6. Conclusion

A combination of factors including unsafe pesticide practice and psychosocial stressors are related to the suicidal behaviour. A multipronged approach linking the interests of public health, mental health, and agriculture is appropriate for serving the shared interest of all three agendas better than each segregated. Intersectoral programmes are needed to link the interests of the agricultural sector, the GPs, the health sector, and the community to prevent DSH and suicide in Namkhana block, as well as the morbidity and mortality of accidental pesticide poisoning. The role of the agricultural department would typically include promotion of safe pesticide practice, train farmers in alternative methods of pesticide use such as Integrated Pest Management (IPM), generating awareness to purchase limited quantities of pesticide, the required amount only, and improve storage facilities, promoting awareness about the positive as well as negative impacts of pesticide use on crops, health, and environment, regulating and supervising sale of pesticides in the region, encouraging farmers to visit health centres in case of occupational exposure. The GP has an important role to play in regulating and supervising the sale of pesticides in the block, coordinating with the agricultural department its various activities and ensuring that the KPS performs his regular duties, and in encouraging supports to those vulnerable to indulging in suicidal behaviour.

The health department should contribute to reducing the morbidity and mortality of pesticide poisoning—whether accidental or intentional—by making cheap antidotes available in the community, improving treatment. Preventing suicide and managing suicidal behaviour also requires sensitising the public to questions about recognising mental illness, which constitutes an important component of suicide prevention, and to recognise the typical socio-cultural contexts in which pesticides are consumed [22]. This study recommends similar studies to be conducted throughout India and elsewhere for suicide prevention and community mental health to distinguish common and distinctive features of suicidal behaviours that local programmes should be aware of.

This community household survey examined practical features and contexts of suicide well beyond rates and psychiatric diagnosis. Findings highlight the need for intersectoral programmes that combine activities to minimise pesticide hazard and recognise the typical contexts in which DSH and suicide occur.

## Figures and Tables

**Table 1:** Distribution of pesticide storage (*n* = 1191*).

Inside the house (*n* = 847)	Yes	%	Outside the house (*n* = 344)	Yes	%
Locked Box	232	**27.3**	Within the courtyard	111	**32.3**
Confidential	248	**29.3**	In the agricultural field	213	**61.9**
Out of reach of children	643	**75.9**	Others	20	**5.7**

*Table includes the 70 households that store pesticides both inside as well as outside the house.

**Table 2:** Cross tabulation of respondents' and investigators' classification of safe storage of pesticide (*n* = 1221).

Respondent assessed		Investigator assessed			Total	
	Safe	%	Unsafe	%	*n*	%
Safe	480	(99.4)	594	(80.5)	1074	(88.0)
Unsafe	3	(0.6)	144	(19.5)	147	(12.0)

Total	483	(39.5)	738	(60.4)	1221	(100.0)

**Table 3:** Sources of information about ill-effects of pesticide on crops and health as reported by households using pesticides (*n* = 1221).

Sources of information**	Crops *n* = 320	Health *n* = 452
	(26.2%)*	(37.0%)*
Agricultural department	27 (8.4)	22 (4.9)
Fellow farmer	113 (35.3)	238 (52.7)
Media	25 (7.8)	39 (8.6)
Gram Panchayats	1 (0.3)	3 (0.7)
Personal experience	218 (68.1)	287 (63.5)
Pesticide company	9 (2.8)	15 (3.3)
Pesticide shop	63 (19.7)	106 (23.5)
Others	8 (2.5)	9 (2.0)

*Percentage is with reference to households using pesticides (*n* = 1221).

**Individuals responded to more than one category.

**Table 4:** Annual overall and pesticide-related DSH and suicide rates per 100,000 population.

Items	All means (including Pesticide)				Pesticide	
	Female	Male	Total	Female	Male	Total
Population	5182	5445	10627	5182	5445	10627
DSH (*n*)*	94	42	136	51	33	84
Rate**	**362.8**	**154.3**	**256.0**	**196.8**	**121.2**	**158.1**
(CI)***	(293.3–443.8)	(111.2–208.5)	(215.0–302.4)	(146.6–258.7)	(83.5–170.2)	(126.2–195.5)
Suicide (*n*)*	25	22	47	23	16	39
Rate**	**96.5**	**80.8**	**88.4**	**88.8**	**58.8**	**73.4**
(CI)***	(62.5–142.4)	(50.7–122.3)	(65.0–117.6)	(56.3–133.2)	(33.6–95.4)	(52.2–100.3)

*n = number of events in 5 years. **Rate is calculated per year. ***The confidence interval was set at 95.0%.
